# Exploring Epidemiological Aspects, Distribution of WHO Maternal Near Miss Criteria, and Organ Dysfunction Defined by SOFA in Cases of Severe Maternal Outcome Admitted to Obstetric ICU: A Cross-Sectional Study

**DOI:** 10.1155/2018/5714890

**Published:** 2018-11-13

**Authors:** Antonio Francisco Oliveira Neto, Mary Angela Parpinelli, Maria Laura Costa, Renato Teixeira Souza, Carolina Ribeiro do Valle, José Guilherme Cecatti

**Affiliations:** Department of Obstetrics and Gynecology, University of Campinas (UNICAMP), Campinas, SP, Brazil

## Abstract

**Objective:**

To explore the epidemiological aspects, to describe the frequency and distribution of WHO maternal near miss (MNM) criteria and the presence of organ dysfunction and failure measured by the maximum SOFA (Sequential Organ Failure Assessment) score (SOFA max) in cases of severe maternal outcome (SMO).

**Methods:**

In an observational cross-sectional study performed between January 2013 and December 2015, 279 pregnant or postpartum women were admitted to an obstetric ICU (intensive care unit) in Brazil. MNM, maternal death (grouped as SMO), and potentially life-threatening conditions (PLTC) were defined according to WHO criteria. For categorical variables, a descriptive analysis was carried out. Frequency and distribution of WHO criteria, organ dysfunction, or failure defined by SOFA max were performed.

**Results:**

WHO criteria identified 65 SMO and 214 PLTC. Management criteria were present in 58/65 (89.2%) while 61/65 (93.8%) of SMO cases had dysfunction or failure by SOFA.

**Conclusions:**

The systematic evaluation of the organic function by SOFA max score identified the presence of organic dysfunction or failure in almost all SMO cases. Management criteria were present in all MD cases. Our results indicate the need for new studies evaluating the parameterization of the WHO laboratory criteria for values compatible with the definition of organic dysfunction by the SOFA to identify MNM.

## 1. Introduction

The concept of severe maternal outcome (SMO) is used to describe a woman who died or survived a life-threatening condition (LTC) during pregnancy or childbirth or in the first 42 days postpartum [1]. Maternal near miss (MNM) identifies a group of women who are survivors of an LTC [1, 2]. Due to its higher prevalence and diverse characteristics in common with cases of maternal death (MD) [3, 4], the study of MNM cases has been shown to complement MD studies. MNM cases can provide useful information concerning the identification of delays, failures, and successes in the care of pregnant or postpartum women [5, 6].

Accurate definition of an LTC is the first essential step in understanding the factors contributing to SMO [1, 7]. It is well-known that organ dysfunction is one of the major determining factors of outcome [8]. Organ function is understood to be altered and homeostasis is only maintained by therapeutic interventions [9]. Criteria based on the evaluation of organ function are considered the gold standard to detect SMO cases [1, 2].

To make the identification of MNM uniform, WHO proposed a multiple approach to the operational definition of the concept of LTC by means of identifying clinical and laboratory criteria for organ function assessment and management criteria consisting of life support procedures and/or interventions that are not usually required during a normal pregnancy or postpartum period [1, 10].

Although WHO MNM criteria are capable of identifying all SMO cases [11, 12], recent studies conducted in high- and middle-income countries have questioned the sensitivity of organ function criteria as a tool for the definition of LTC [13, 14]. According to these authors, the WHO based organ function criteria can underestimate the severity of cases, and it may be missing in up to 35% of maternal deaths (MD) and although the management-based criteria are able to identify almost all cases of SMO, these criteria are not suitable for comparison studies [13, 14].

Conceptually, MNM has epidemiological, clinical, and pathophysiological characteristics that are similar to MD cases. The operational definition of near miss cases by WHO brought uniformity to studies of severe maternal morbidity (SMM), which contributed to a better understanding of the chain of events that determine the outcome, allowing the evaluation and identification of its determinants in different regions or countries [1–6]. The aim of this study is to explore the epidemiological aspects, to describe the rate and distribution of WHO MNM criteria and the presence of organ dysfunction or failure measured by the SOFA (Sequential Organ Failure Assessment) score in SMO cases admitted to an obstetric intensive care unit (ICU).

## 2. Materials and Methods

This is a cross-sectional study performed at the Women's Hospital, Brazil. That is a public teaching hospital of the University of Campinas, where around 2,800 deliveries are performed annually. Included in the study were all pregnant and/or postpartum women within 42 days of childbirth admitted to the obstetric ICU, where they stayed for at least 24 hours, from January 1^st^, 2013, to December 31^st^, 2015.

The ICU manages obstetric cases requiring tertiary and secondary levels of care, according to admission criteria of the American College of Critical Care Medicine and the Society of Critical Care [15]. The study did not perform interventions other than routine care and was approved by the Institutional Review Board (letter of approval number 1,046,997 from May 4^th^, 2015).

Cases were identified in the hospital inpatient electronic system. Medical charts were selected and reviewed. Primary complications that justified admission were classified as direct obstetric, indirect obstetric, or clinical-surgical causes. Direct obstetric morbidities were categorized as hypertensive, hemorrhagic, and infectious. In addition, identification of diseases specific for each category was made. Indirect obstetric morbidities were identified by comorbidity and categorized into systemic inflammatory, endocrinological, hematological, cardiopulmonary, neurological, and gastrointestinal.

**Figure 1:**
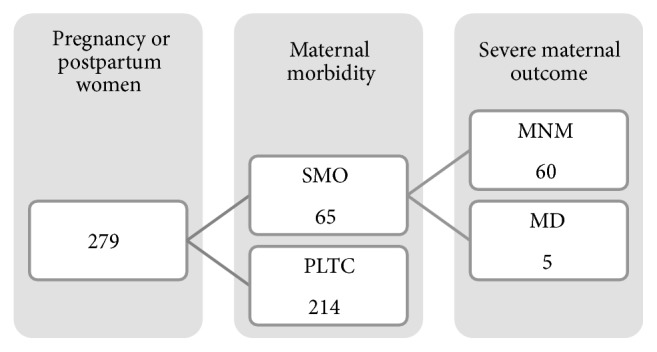
Flowchart for the identification of cases of severe maternal morbidity using the WHO criteria.

The identification of SMO (MD and MNM) and potentially life-threatening conditions (PLTC) followed criteria established by WHO [1]. Criteria for the definition of an LTC are distributed into clinical, laboratory, and management criteria that represent a loss of body homeostasis. The presence of one or more of these criteria identifies an SMO (Figure 1).

The variables selected were maternal age, type of ICU admission (elective or emergency), the source of hospital admission, and medical history. The morbid condition that motivated admission was defined on the first day of hospitalization and should reflect its primary reason. When multiple diagnoses were present, the most severe diagnosis and worst prognosis were considered.

For each admission, a group of variables was collected to characterize the admission, procedures, or interventions performed in the ICU. The variables were used to calculate the SOFA score [8]. This score determines the functioning of the respiratory, hematological, hepatic, cardiovascular, neurological, and renal systems. The function of each of the six systems is graded from 0 to 4. A zero score is normal and scores 1, 2, 3, and 4 represent progressive degrees of dysfunction. Organ dysfunction is defined as a SOFA ≥ 1 and ≤ 2 and failure ≥ 3 and ≤ 4 points. The worst results found for each of the six systems throughout ICU admission are the maximum SOFA (SOFA max) [16].

Since arterial blood gas sampling is not routinely collected in all pregnant or postpartum patients admitted to the ICU, oxygen arterial pressure (PaO_2_) records were missing in some cases. In this situation, oxygen saturation was assumed (Sat O_2_) and obtained by pulse oximeter readings to calculate the SaTO_2_/FiO_2_ ratio that was used by the scores [17]. For cases in which evaluation of the level of consciousness by the Glasgow coma scale (GCS) was compromised due to residual effects of anesthetics in the postoperative period, or by the use of continuous sedation, normality was the parameter used, i.e., GCS of 15 [18].

Collected data was transferred and stored in an electronic database in the web REDCap (Research Electronic Data Capture) platform. SMO cases were descriptively evaluated according to the distribution of their sociodemographic, obstetric, and admission characteristics. Then, the composition of different groups of morbidity causes was evaluated in women with SMO and compared to the remaining PLTC group. The distributions of causes of morbidity as direct or indirect were compared in the groups of SMO and PLTC and the significance of differences was assessed using Chi-square test. Criteria defining the LTC were comparatively evaluated between MD and MNM cases. Finally, the SOFA max was evaluated in a grouped and decomposed manner to determine the existence of organ dysfunction and/or failure for each of the six systems evaluated, in a comparison between MD and MNM.

## 3. Results

During the 36-month study period, there were 8,077 deliveries with 8,065 live births. Two hundred and seventy-nine pregnant or postpartum women within 42 days of childbirth were admitted to the obstetric ICU. Of these, 65 progressed to SMO (5 MD and 60 MNM). The ratio of MNM (MNMR=MNM/1,000 LB) was 7.4/1,000 LB and the ratio case/fatality was 13 MNM: 1 MD.

Among the SMO cases, 11/65 pregnant or postpartum women within 42 days of childbirth were adolescents and one-fourth of the women (17/65) were aged 35 or older. The highest percentage of admissions occurred in the postpartum period (60% or 39/65 cases). Cesarean delivery was the route of pregnancy resolution in 74% of SMO cases. A history of chronic morbidity was present in 40 of the total number of cases (61.5%) (Table 1).

Morbid conditions that motivated admissions were distributed as direct and indirect obstetric causes, by subgroups and by specific causes as observed in Table 2. The subgroup of hemorrhagic morbidities was responsible for the largest proportion of SMO (20 out of 31 cases). Indirect causes motivated 32.7% of admissions of cases with PLTC and were responsible for 52.3% of SMO, a significant difference (p=0.006). Regarding the five deaths, one was due to direct cause (eclampsia and PRES, posterior reversible encephalopathy syndrome) and four were due to indirect causes (pulmonary thromboembolism, systemic lupus erythematosus, bacterial pneumonia, and hemorrhagic shock in a patient with portal hypertension).

Management criteria were identified in 100% of deaths and in 53/60 MNM cases (88.3%). Only combined laboratory and management criteria were capable of identifying the 60 MNM cases and five deaths. The most frequent criteria recorded were the use of vasoactive drugs with 40 events (28.7%) and mechanical ventilation with 30 events (21.5%). The distribution and frequency of the MNM criteria may be observed in Table 3.

Organ function determined by SOFA max in a grouped and decomposed manner in six organs is shown in Table 4. All five MD had a failure of one or more organs. Of the 60 MNM cases, SOFA max from six systems was zero (no dysfunction or failure) in four patients (6.7%). In total, organ dysfunction and/or failure by SOFA was recorded in 93.7% of SMO in the ICU.

## 4. Discussion

The exploratory nature of our study allowed us to conclude that a systematic evaluation of the organic function by SOFA max score identified the presence of dysfunction or organic failure in virtually all SMO cases. Most SMO cases were identified by management criteria.

As observed by other researchers, most admissions that originated in hospital units occurred in the postpartum period and cesarean section was the mode of delivery in the majority of women [19–21]. Various authors have observed that there was a significant association between cesarean section and MNM [4–6, 22]. In the current study, cesarean section represents the route of resolution in the vast majority of pregnancies. However, some researchers have questioned this association. The great dilemma remains: whether cesarean delivery is defined as a risk factor for MNM, or whether it is actually a consequence of this condition.

According to other authors, most of the admissions were due to direct obstetric causes, and hypertensive obstetric morbidities were the most frequent [11, 19–21]. In the subgroup of obstetric morbidities of direct causes, the most severe cases were hemorrhagic morbidities. These findings are in agreement with studies indicating that the obstetric hemorrhagic morbidities are more severe and have the greatest mortality in the group of direct causes [20].

In agreement with other researchers, the group of indirect obstetric causes contains very severe cases and a higher number of deaths [19, 20]. In this group, we are concerned with the potentially preventable SMO cases motivated by infectious complications (35%), especially urinary tract infections (7 out of 12 or 58%). Maternal sepsis is directly related to an increased risk of spontaneous abortion, preterm birth, and fetal and maternal death [23, 24]. The use of protocols for screening and treating for antenatal urinary tract infection as a public health policy can contribute to reducing maternal sepsis, especially in low- and middle-income countries [23].

Management criteria were responsible for identifying the majority of cases. The same result was found by researchers investigating ICU admissions of SMM cases in the Netherlands [13] and in Brazil [11] and others who compared results between countries of low- and middle-income with high-income countries [14]. It was also different from the results obtained by another study conducted in Brazil, where laboratory criteria were present in almost 60% of MNM cases [22]. Local characteristics, different morbid conditions that motivated hospital admission, and differences in management protocols may have contributed to these divergent results.

WHO criteria for organ function were recorded in 93.7% of MNM and all MD cases. A similar result was found by researchers investigating maternal ICU admissions [11]. These results were different from those found by studies in which organ dysfunction criteria were missing in 16.6% to 35% of deaths [13, 14]. Organ dysfunction precedes failure and should be understood as the first sign of severity for an unfavorable progression [7, 9, 16]. Organ failure is part of a pathophysiological process that culminates in death [8, 9, 16].

In this study, laboratory criteria were present in 35% of SMO cases. When comparing the variables used by SOFA, WHO laboratory criteria are compatible with the definition of organ failure (SOFA ≥ 3) [8]. The use of these cut-off values increases specificity, although sensitivity for the identification of potentially severe cases (organ dysfunction) is decreased. The use of SOFA to identify MNM is good as clinical criteria are the gold standard to measure organ dysfunction and this score is routinely used in clinical settings, as general ICU. However, the presence of mild dysfunction by SOFA (score 1-2) may be interpreted as minor damage and, regarding obstetrical conditions, with their particular evolution and presentation, this may be a limitation that could allow delays in the provision of care.

Management criteria and not laboratory criteria would be useful to identify SMO because they are more related to organ failure. The most frequent criteria recorded in the study, mechanical ventilation and/or vasoactive drugs, are the best examples for this statement. Even in ARDS (Acute Respiratory Distress Syndrome) cases, laboratory criteria for oxygenation as defined by WHO (PaO_2_/FiO_2_ < 200) will be missing, for instance, in a patient under mechanical ventilation with mild ARDS (PaO_2_/FiO_2_ > 200 ≤ 300). For the variable use of vasoactive drugs, the WHO does not establish any other criteria for stratification of severity such as blood pressure levels or type of drug (vasodilator or vasoconstrictor) used, which could limit its usefulness for this purpose. This should be better addressed and possibly changed. Hypothetically a situation when nitroprussiate is used for the hypertensive emergency condition for a short period would have the same value as the prolonged use of vasoconstrictive drugs due to septic shock, and in fact, they are completely different clinical scenarios also with distinct prognosis.

Management criteria demand a hospital and laboratory structure of higher complexity [2] and although they are able to identify almost all cases of SMO, they are however influenced by bed availability and different criteria used for ICU admission, different local protocols for transfusing blood products, and use of mechanical ventilation or vasoactive drugs, for example [11, 13, 14, 22].

Four cases without dysfunction or failure determined by SOFA were defined as MNM by the presence of one management criterion in each case (two by the use of vasoactive drugs and one by red packed cell transfusion ≥ 5 units and puerperal hysterectomy). The continuous use of intravenous vasodilators in hypertensive emergencies is not a variable evaluated by SOFA [8]. On the other hand, the performance of puerperal hysterectomy for infectious or hemorrhagic morbidities is a management criterion considered essential for the maintenance of the woman's life. We believe that the definition of SMO based only in a red packed cell transfusion (≥ 5 units) should be analyzed and criteria based on the clinical classification of the degree of shock [24] and/or the shock index (SI) [25] should be better considered in future studies.

Our study has limitations that should be taken into account. It is an exploratory retrospective study, conducted in a tertiary hospital from a middle-income country, whose results may not apply to the general population from everywhere. The small number of MNM and MD cases precluded a comparative analysis with statistical power. Hopefully, these points will be instigating new studies on the topic in different populations.

Another concern in using only SOFA criteria to identify SMO cases is that in most of the low- and middle-income countries there is a lack of record's data completeness, so the clinical measurements may be missing, although the management procedures to deal with the dysfunction/failure are less likely to be missing on medical records. It should be emphasized that a prospective standardized surveillance following previously chosen criteria is fundamental. If the SOFA is chosen, the service has to use all parameters for its calculation systematically registered for all cases.

In conclusion, the proportion of direct and indirect obstetric causes of morbidity determining admission to an obstetric ICU is significantly different between cases of PLTC and those more severe, identified as SMO. Organ dysfunction precedes failure and should be understood as the first sign of severity for an unfavorable progression. The SOFA max identified the presence of dysfunction or organic failure in almost all SMO cases and can therefore be used as a maker to identify these more severe cases. Management criteria were responsible for identifying the majority of MNM cases. Our results indicate the need for new studies evaluating the parameterization of the WHO laboratory criteria for values compatible with the definition of organic dysfunction by the SOFA to better identify MNM cases.

## Figures and Tables

**Table 1:** Sociodemographic, obstetric, and clinical characteristics of women with severe maternal outcome (SMO).

**Characteristics**	**SEVERE MATERNAL OUTCOME**	
	**N**	%
**Age (years)**		
≤ 19	11	16.9
> 20 - 34	37	56.9
≥ 35	17	26.2

**Parity**		
Nulliparous	40	61.5
≥ 1	25	38.5

**Gestational age at delivery (week) ** ^**a**^		
≤ 34	21	40.4
> 34	31	59.6

**Any medical conditionmedical history**	40	61.5

**Mode of delivery ** ^**b**^		
Vaginal	13	26
Cesarean section	37	74

**Admission source**		
Emergency room	10	15.4
Hospital wards	45	69.2
Transferred from another hospital	10	15.2

**Status at admission**		
Postpartum	39	60
Pregnancy	26	40

**Length of ICU stay (days)**		
1-2	20	30
≥ 3	45	69.2

**Total**	**65**	100

^a.^n.a for 13 cases, ^b^n.a for 15 cases (14 women were admitted to the ICU during pregnancy, were treated, and were discharged still pregnant, while one woman died still pregnant).

**Table 2:** Distribution of causes of admission in the obstetric intensive care unit according to the maternal outcome as defined by WHO*∗*.

**Causes – N (**%**)**	**SMO**	**PLTC**
	**N (**%**)**	**N (**%**)**
**Direct obstetric - 175 (62.7**)*∗*	**31 (47.7)**	**144 (67.3)**
(i) **Hypertensive - **135 (77.1)	8 (25.8)	127 (88.2)
Preeclampsia	4	99
Eclampsia	2	4
HELLP syndrome	2	22
Hypertensive crisis	0	2
(ii) **Hemorrhage** - 35 (20)	20 (64.5)	15 (10.4)
Postpartum	19	13
Antepartum	1	2
(iii) **Infectious** - 4 (2.3)	2 (6.5)	2 (1.4)
Chorioamnionitis	0	1
Endometritis	2	0
Septic abortion	0	1
(iv) **Peripartum cardiomyopathy - **1 (0.6)	1 (3.2)	0 (-)

**Indirect obstetric - 104 (37.3)**	**34 (52.3)**	**70 (32.7)**
(i) **Infectious disease - **35 (33.6)	12 (35.2)	23 (32.9)
Urinary tract	7	11
Pneumonia	5	5
Dengue	0	2
Others	0	5
(ii) **Inflammatory and endocrine disease - **7 (6.7)	3 (8.8)	4 (5.7)
SLE	2	1
Adrenal tumor	1	1
Addison syndrome	0	1
Diabetic ketoacidosis	0	1
(iii) **Hematologic disease - **8 (7.7)	4 (11.8)	4 (5.7)
TP	2	1
Hemolytic anemia	2	3
(iv) **Heart and pulmonary disease** - 31 (29.9)	8 (26.4)	23 (32.9)
Pulmonary edema	2	8
Pulmonary embolism	1	3
Severity acute asthma	2	0
TRALI	1	0
SIRS	1	0
Myasthenic crisis	1	0
other	0	12
(v) **Neurological disease** - 13 (12.5)	4 (11.8)	9 (12.8)
Seizure (no eclampsia)	2	8
Intracranial hypertension/tumor	1	0
Hemorrhagic stroke	1	0
other	0	1
(vi) **Gastrointestinal tract disease** - 10 (9.6)	3 (8.8)	7 (10)
Ogilvie's syndrome	1	0
Portal hypertension/hypovolemic shock	1	1
(vii) Acute pancreatitis	1	0
other	0	6

**Total**	**65 (100)**	**214 (100)**

SMO= severe maternal outcome, PLTC= potentially life-threatening condition, HELLP= hemolysis, elevated liver enzymes, low platelet,

SLE= systemic lupus erythematosus, TP= thrombocytopenic purpura, TRALI= transfusion-related acute lung injury, and

SIRS= systemic inflammatory response syndrome.

**∗**According to WHO definition (Say et al., 2009, [2]).

**∗** **∗**Chi-square= 7.37, p=0.006 (direct X indirect causes).

**Table 3:** Frequency and distribution of maternal near miss criteria according to the maternal outcome.

**WHO Life-threatening conditions** **∗**		**MD**	**MNM**
	**N [Events]**	**N [Events]**	**N [Events]**
**Clinical **	**10 [** **10]**	**3 [** **3]**	**7 [** **7]**

**Respiratory**			
Severe tachypnea (RR > 40 bpm) or bradypnea (RR < 6 bpm)		1	0

**Circulatory**			
Cardiac arrest		0	1

**Renal and Hepatic**			
Oliguria		1	1
Jaundice in the PE		0	3

**Neurological**			
Glasgow < 10 (lasting ≥ 12h)		1	0
Stroke		0	1

**Coagulation/Hematological**			
Failure to form clots		0	1

**Laboratory**	**23 [29** **]**	**3 [** **6]**	**20 [** **23]**
PaO2 /FiO2 < 200mmHg		3	12
Creatinine ≥ 300*μ*mol/L or ≥ 3,5mg/dL		1	2
Bilirubin ≥ 100*μ*mol/L or ≥ 6,0mg/dl		0	2
Thrombocytopenia acute (< 50.000 plt/ml)		1	5
Lactate > 5 mmol		0	1
pH < 7.1		1	1

**Management**	**58 [** **100]**	**5 [** **16]**	**53 [** **84]**
Use of vasoactive drugs		5	35
Hysterectomy		0	9
Blood transfusion ≥5 units		1	14
Invasive mechanical ventilation > 1h		5	25
Hemodialysis		1	0
CPR		4	1

**Combined (clinical and laboratory)**		**4**	**23**

**Combined (clinical and management)**		**5**	**53**

**Combined (laboratory and management)**		**5**	**60**

**Total**	**65 [** **139]**	**5 [** **25]**	**60 [** **114]**

MD= maternal death, MNM= maternal near miss, RR= respiratory rate, PE= preeclampsia, PaO2= arterial oxygen tension, PLT= platelets, FiO2= fraction of inspired oxygen, pH= hydrogen ion concentration, CPR= cardiopulmonary resuscitation.

**∗** According to WHO definition (Say et al., 2009, reference [2])

**Table 4:** Evaluation of organ function by the SOFA (Sequential Organ Failure Assessment) maximum score during intensive care unit stay in cases of severe maternal outcome.

**SOFA score**	SEVERE MATERNAL OUTCOME	
	**MD**	**MNM**
	**N (**%**)**	**N (**%**)**
**SOFA max**		
0 (no dysfunction)	0	4 (6.7)
≥ 1 and ≤ 2 (dysfunction)	0	24 (40)
≥ 3 and ≤ 4 (failure)	5 (100)	32 (53.3)

SOFA max Respiratory		
0	0	19
≥1 and ≤ 2	2	29
≥ 3	3	12
SOFA max Cardiovascular		
0	0	28
≥1 and ≤ 2	0	12
≥ 3	5	20
SOFA max Neurological		
0	3	50
≥1 and ≤ 2	0	10
≥ 3	2	0
SOFA max Renal		
0	2	52
≥1 and ≤ 2	2	6
≥ 3	1	2
SOFA max Hepatic		
0	5	48
≥1 and ≤ 2	0	10
≥ 3	0	2
SOFA max Coagulation		
0	2	42
≥1 and ≤ 2	2	13
≥ 3	1	5

**Total**	**5 (100)**	**60 (100)**

MD: maternal death, MNM: maternal near miss, and SOFA: Sequential Organ Failure Assessment.

SOFA max (SOFA maximum): 0 to 4 points, 0= normal, ≥1 and ≤ 2= dysfunction, and ≥ 3= failure.

## Data Availability

The data used to support the findings of this study are available from the corresponding author upon request.

## References

[1] World Health Organization (2011). *The near-miss approach for maternal health. Evaluating the quality of care for severe pregnancy complications*.

[2] Say L., Souza J. P., Pattinson R. C. (2009). for the WHO working group on Maternal Mortality and Morbidity classifications. *Best Pratice Research Clinical Obstetrics & Gynaecology*.

[3] Tunçalp Ö., Hindin M. J., Souza J. P., Chou D., Say L. (2012). The prevalence of maternal near miss: a systematic review. *BJOG: An International Journal of Obstetrics & Gynaecology*.

[4] Purandare C. N. (2013). Maternal near miss review: A way forward. *The Journal of Obstetrics and Gynecology of India*.

[5] Akrawi V. S., Al-Hadithi T. S., Al-Tawil N. G. (2017). Major determinants of maternal near-miss and mortality at the maternity teaching hospital, Erbil city, Iraq. *Oman Medical Journal*.

[6] Mekango D. E., Alemayehu M., Gebregergs G. B., Medhanyie A. A., Goba G. (2017). Determinants of maternal near miss among women in public hospital maternity wards in Northern Ethiopia: A facility based case-control study. *PLoS ONE*.

[7] Clark S. L., Hankins G. D. V. (2012). Preventing maternal death: 10 clinical diamonds. *Obstetrics & Gynecology*.

[8] Vincent J.-L., Moreno R., Takala J. (1996). The SOFA (Sepsis-related Organ Failure Assessment) score to describe organ dysfunction/failure. *Intensive Care Medicine*.

[9] Fink M. P., Evans T. W. (2002). Mechanisms of organ dysfunction in critical illness: Report from a Round table Conference held in Brussels. *Intensive Care Medicine*.

[10] Souza J., Tunçalp Ö., Vogel J. (2014). Obstetric transition: the pathway towards ending preventable maternal deaths. *BJOG: An International Journal of Obstetrics & Gynaecology*.

[11] Cecatti J. G., Souza J. P., Oliveira Neto A. F. (2011). Pre-validation of the WHO organ dysfunction based criteria for identification of maternal near miss. *Reproductive Health*.

[12] Souza J. P., Cecatti J. G., Haddad S. M. (2012). The WHO maternal near-miss approach and the maternal severity index model (MSI): tools for assessing the management of severe maternal morbidity. *PLoS ONE*.

[13] Witteveen T., De Koning I., Bezstarosti H., Van Den Akker T., Van Roosmalen J., Bloemenkamp K. W. (2016). Validating the WHO Maternal Near Miss Tool in a high-income country. *Acta Obstetricia et Gynecologica Scandinavica*.

[14] Witteveen T., Bezstarosti H., de Koning I. (2017). Validating the WHO maternal near miss tool: Comparing high- and low-resource settings. *BMC Pregnancy and Childbirth*.

[15] Nates J. L., Nunnally M., Kleinpell R. (2016). ICU admission, discharge, and triage guidelines: A framework to enhance clinical operations, development of institutional policies, and further research. *Critical Care Medicine*.

[16] Moreno R., Vincent J.-L., Matos R. (1999). The use of maximum SOFA score to quantify organ dysfunction/failure in intensive care. Results of a prospective, multicentre study. *Intensive Care Medicine*.

[17] Pandharipande P. P., Sanders N., St Jacques P., Ely E. W., Shintani A. (2006). Calculating SOFA scores when arterial blood gasses are not available: validating SpO2/FiO2 ratios in the SOFA scores. *Critical Care Medicine*.

[18] Breslow M. J., Badawi O. (2012). Severity Scoring in the Critically Ill. *CHEST*.

[19] Chantry A. A., Deneux-Tharaux C., Bonnet M., Bouvier-Colle M. (2015). Pregnancy-Related ICU Admissions in France. *Critical Care Medicine*.

[20] Vasquez D. N., Das Neves A. V., Vidal L. (2015). for the ProPOC Study Group, Characteristics, outcome, and predictability of critically ill obstetric patients: a multicenter prospective cohort study. *Critical Care Medicine*.

[21] Jain S., Guleria K., Suneja A., Vaid N. B., Ahuja S. (2016). Use of the Sequential Organ Failure Assessment score for evaluating outcome among obstetric patients admitted to the intensive care unit. *International Journal of Gynecology and Obstetrics*.

[22] Oliveira L. C., Da Costa A. A. R. (2015). Maternal near miss in the intensive care unit: Clinical and epidemiological aspects. *Revista Brasileira de Terapia Intensiva*.

[23] Lee A. C., Quaiyum M. A., Mullany L. C. (2015). Screening and treatment of maternal genitourinary tract infections in early pregnancy to prevent preterm birth in rural Sylhet, Bangladesh: a cluster randomized trial. *BMC Pregnancy and Childbirth*.

[24] American College of Surgeons (2012). *Advanced Trauma Live Support – ATLS*.

[25] Nathan H. L., Cottam K., Hezelgrave N. L. (2016). Determination of Normal Ranges of Shock Index and Other Haemodynamic Variables in the Immediate Postpartum Period: A Cohort Study. *PLoS ONE*.

